# Evaluation of Remdesivir for Mildly to Moderately Ill Patients with COVID-19: A Single-Arm, Single-Center, Retrospective Study

**DOI:** 10.3390/medicina58081007

**Published:** 2022-07-27

**Authors:** Motoyasu Miyazaki, Ryoko Yanagida, Akio Nakashima, Koichi Matsuo, Norihiro Moriwaki, Masanobu Uchiyama, Yota Yamada, Hitomi Hirata, Hisako Kushima, Yoshiaki Kinoshita, Hiroshi Ishii, Osamu Imakyure

**Affiliations:** 1 Department of Pharmaceutical and Health Care Management, Faculty of Pharmaceutical Sciences, Fukuoka University, Fukuoka 814-0180, Japan; anakashima@fukuoka-u.ac.jp (A.N.); ko1matsuo@fukuoka-u.ac.jp (K.M.); imakyure@fukuoka-u.ac.jp (O.I.); 2 Department of Pharmacy, Fukuoka University Chikushi Hospital, Chikushino 818-8502, Japan; 28574988a@gmail.com (R.Y.); muchiyama@fukuoka-u.ac.jp (M.U.); yota0114@fukuoka-u.ac.jp (Y.Y.); h.hirata.cd@adm.fukuoka-u.ac.jp (H.H.); 3 Department of Pharmacy, Fukuoka University Hospital, Fukuoka 814-0180, Japan; nmoriwaki@fukuoka-u.ac.jp; 4 Department of Respiratory Medicine, Fukuoka University Chikushi Hospital, Chikushino 818-8502, Japan; hkushi@fukuoka-u.ac.jp (H.K.); y3kinoshita@fukuoka-u.ac.jp (Y.K.); hishii@fukuoka-u.ac.jp (H.I.)

**Keywords:** coronavirus, COVID-19, remdesivir, oxygen therapy

## Abstract

*Background and Objectives*: Remdesivir (RDV) is the first antiviral agent approved in Japan for the treatment of coronavirus disease 2019 (COVID-19). The aim of our study was to assess the efficacy and safety of RDV treatment in mildly to moderately ill patients with COVID-19. *Materials and Methods:* A single-center, retrospective study was performed in Fukuoka University Chikushi Hospital. Patients admitted to our hospital from June to October 2021 for RDV treatment against COVID-19 were enrolled. The primary end point was clinical status on days 10 and 14, using a 6-point ordinal scale ranging from death (category 6) to discharge (category 1). Adverse events were assessed and graded using the Japanese version of Common Terminology Criteria for Adverse Events (CTCAE) v5.0. *Results*: In total, 47 COVID-19 patients receiving RDV treatment were assessed during the study period. Thirty-four (72.3%) out of 47 patients required oxygen therapy. Out of these 34 patients, 30 (88.2%) showed a 2-point clinical improvement on day 14 after RDV was initiated. Serum alanine aminotransferase levels were elevated in three patients (6.4%) (CTCAE Grade 3) and neutropenia was detected in one patient (2.1%) out of the 47 patients. *Conclusions*: RDV may be highly effective, with good safety profiles, in patients with COVID-19 requiring oxygen therapy.

## 1. Introduction

Coronavirus disease 2019 (COVID-19) is a public health emergency and rapidly spread worldwide from its first discovery in December 2019. COVID-19 was declared a pandemic by the World Health Organization on 11 March 2020 [1]. In Japan, on 16 January 2020, the government announced the first COVID-19 infected individual in Kanagawa prefecture, who had a history of traveling to Wuhan, China. Thus far in Japan (April 2022), there have been six major “infection waves”, and the cumulative number of infected and dead patients is still increasing.

Remdesivir (RDV) was approved as the first medicine for COVID-19 treatment in Japan on 7 May 2020 and could only be used for critically ill patients requiring mechanical ventilation. However, some clinical trials reported that RDV treatment was not associated with statistically significant clinical benefits in severe inpatients with COVID-19 [2,3,4]. Based on these results, from 7 January 2021, indications for RDV were expanded to patients with moderate to critical pneumonia who did not necessarily require oxygen administration. RDV is a direct-acting antiviral agent that inhibits viral RNA synthesis. Severe acute respiratory syndrome coronavirus 2 (SARS-CoV-2) invades the cell by binding the spike protein on the surface of the virus to the ACE-2 receptor of the host cell and proliferates by replicating the viral genome inside the cell [5]. When RDV is taken into cells, it undergoes intracellular metabolism and is converted to RDV triphosphate (RDV-TP), which is an active metabolite of the nucleoside triphosphate type. RDV-TP, as an analog of adenosine triphosphate, is incorporated into the RNA strand replicated by the RNA-dependent RNA polymerase of SARS-CoV-2 and suppresses viral replication by inhibiting RNA synthesis in cells [6,7].

Olender et al. reported that RDV was associated with significantly higher clinical recovery rates on day 14 and lower 28-day mortality rates when compared with standard-of-care treatments in hospitalized patients with COVID-19 [8,9]. Mozaffari et al. also reported that in a large, multicenter, observational cohort study, including more than 100,000 patients, RDV treatment upon hospital admission was associated with significant survival benefits when compared with a non-RDV treatment group [10]. RDV is approved for patients with a wide range of severity, from mild to severe COVID-19. Although oral antiviral drugs such as molnupiravir and nirmatrelvir/ritonavir can be used in mildly to moderately ill patients who do not require oxygen therapy, molnupiravir has contraindications for pregnant women, and nirmatrelvir/ritonavir, which is a strong CYP3A4 inhibitor, has many contraindicated medications that cannot be used in combination. [11]. RDV is the only approved antiviral drug in Japan that can be used in patients with moderate to severe COVID-19, and its efficacy and safety have been reported worldwide. However, few reports have investigated the efficacy and safety profiles of RDV in real-world clinical practice in Japan, and also few reports have investigated associated clinical factors. Data collection in clinical practice will significantly contribute to future clinical approaches for COVID-19 patients. Our aim was to investigate the clinical efficacy and safety profiles in COVID-19 patients receiving RDV treatment in a single university hospital setting in Japan.

## 2. Materials and Methods

### 2.1. Study Setting and Patients

Fukuoka University Chikushi Hospital (Chikushino, Japan) was approved as a regional medical care support hospital by the prefectural governor in April 2007. Our hospital was certified as a priority medical institution that mainly accepted moderate COVID-19 patients; when/if the patient symptoms became severe during hospitalization, patients were transferred to an advanced medical institution.

**Figure 1:**
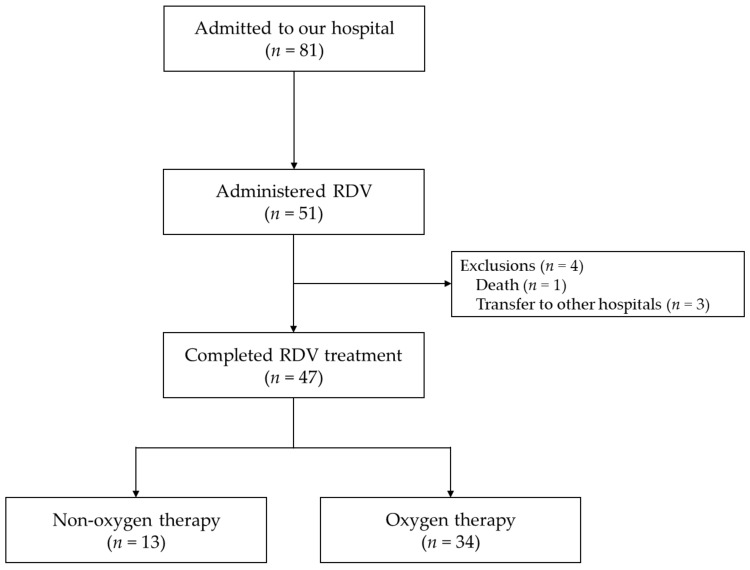
The study flowchart.

Since all COVID-19 patients admitted to our hospital are eligible for antiviral treatment, a placebo-controlled study was not possible. In addition, there is no approved antiviral other than RDV that can be used for moderately ill patients requiring oxygen therapy in Japan; there was no other control group to which the efficacy of RDV was compared. Therefore, we conducted a single-arm, single-center, retrospective study of RDV at our hospital. In total, 81 COVID-19 patients were admitted between June and October 2021 (during the fifth COVID-19 infection wave in Japan). Of these, patients who required RDV treatment were included in this study (Figure 1). Patients who died or transferred to other hospitals due to COVID-19 aggravation during RDV treatment, those under 15 years, and those receiving other COVID-19 treatments prior to RDV were excluded. The study was conducted in accordance with the Declaration of Helsinki and approved by the Fukuoka University Medical Ethics Review Board (C21-12-003).

### 2.2. Clinical Characteristics

Patient data were collected from electronic medical records and reviewed retrospectively. Clinical characteristics included age, gender, height, weight, body mass index (BMI), prior COVID-19 vaccination, smoking history, history of adverse events, comorbidities, laboratory findings, and concomitant drug usage. Comorbidities included hypertension, diabetes mellitus (DM), dyslipidemia, cerebrovascular diseases, asthma, and malignant tumors. Concomitant drugs included dexamethasone, baricitinib, heparin, and antibiotics.

### 2.3. Study Outcomes

The clinical efficacy of RDV for COVID-19 patients was assessed by clinical improvement assessment within 10 or 14 days after RDV administration. Clinical improvement was defined as a 1- or 2-point reduction when compared with patient admission status on a 6-point ordinal scale, as described previously [2,12]. Briefly, the 6-point scale included the following: death = 6; hospital admission for extracorporeal membrane oxygenation and/or invasive mechanical ventilation = 5; hospital admission for non-invasive ventilation (NIV) and/or high flow oxygen therapy (high flow nasal cannula: HFNC) = 4; hospital admission for oxygen therapy (but not requiring NIV/HFNC) = 3; hospital admission but not requiring oxygen therapy = 2; and discharge or having reached discharge criteria (defined as clinical recovery, i.e., fever, respiratory rate, and oxygen saturation returned to normal, and cough relief, all of which were maintained for at least 72 h) = 1. Adverse events were assessed and graded for the 47 patients undergoing RDV treatment using the Japanese version of Common Terminology Criteria for Adverse Events (CTCAE) v5.0.

### 2.4. Statistical Analysis

Binary variables were expressed as proportions, while continuous variables were expressed as medians and interquartile ranges (IQRs). Differences in continuous variables between groups were evaluated using the Wilcoxon rank-sum test, and differences in categorical variables were evaluated using chi-square or Fisher’s exact tests. We divided our study population into two groups based on oxygen therapy: non-oxygen therapy group (*n* = 13) and oxygen therapy group (*n* = 34) (Figure 1). We then compared patient characteristics between groups. Statistical analyses were performed using JMP^®^ 14 (SAS Institute Inc., Cary, NC, USA). A *p* < 0.05 value was considered statistically significant.

## 3. Results

### 3.1. Clinical Characteristics of Patients Completing RDV Treatment

A total of 51 patients required RDV treatment during the study period. Patients who died (*n* = 1) or transferred to other hospitals (*n* = 3) due to COVID-19 aggravation during RDV treatment were excluded. In total, 47 COVID-19 patients treated with RDV were included for RDV efficacy and safety evaluations (Figure 1).

During the study period, 47 COVID-19 patients completed RDV treatment. We observed that 72% (*n* = 34) of the patients were at disease severity point = 3 (hospitalized, requiring supplemental oxygen (but not NIV/HFNC)), the moderately severe category (i.e., oxygen therapy group), and 28% (*n* = 13) were at disease severity point = 2 (hospitalized, but not requiring oxygen therapy), the mildly severe category (i.e., non-oxygen therapy group). Clinical characteristics between groups were compared (Table 1). We observed no differences in clinical characteristics between groups, such as age, gender, BMI, prior vaccination, smoking history, and comorbidities. Patients in the oxygen therapy group had higher values of white blood cells, red blood cells, hemoglobin, and lactate dehydrogenase compared to the patients in the non-oxygen therapy group. All patients requiring oxygen therapy received dexamethasone in combination with RDV.

### 3.2. Outcomes of Patients Completing RDV Treatment

**Figure 2:**
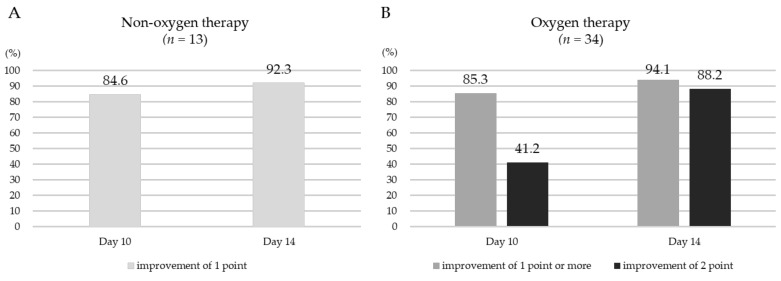
Clinical improvements as assessed by a 6-point scale within 10 or 14 days after remdesivir administration in the non-oxygen therapy group (**A**) and the oxygen therapy group (**B**).

Clinical improvements as assessed by the 6-point scale within 10 or 14 days after RDV administration in COVID-19 patients are shown (Figure 2). In the non-oxygen therapy group (Figure 2A), on the 10th day and 14th day after RDV administration, 84.6% (11/13) and 92.3% (12/13) of patients showed a clinical improvement of 1 point, respectively. In the oxygen therapy group (Figure 2B), on the 10th day after RDV administration, 85.3% (29/34) of patients showed a clinical improvement of 1 point or more, but less than half of the patients did not improve by 2 points. On the 14th day after RDV administration, 94.1% (32/34) of patients showed a clinical improvement of 1 point or more, but four patients (11.8%) did not show a 2-point improvement (Table 2).

### 3.3. Adverse Events in Patients Who Completed RDV Treatment

Adverse events, determined as CTCAE Grade 3 or higher, are shown (Table 3). Serum alanine aminotransferase levels were elevated in three patients (6.4%) and neutropenia was detected in one patient (2.1%) out of 47 patients. Laboratory test values for these patients with adverse events were normalized after RDV treatment. None of the patients had worsened renal function due to RDV administration. No clinically serious adverse events were observed in the study period.

## 4. Discussion

We conducted a single-arm, single-center, retrospective study to assess the efficacy and safety profiles of RDV treatment and found that RDV may be highly effective and safe in mildly to moderately ill patients with COVID-19.

In some previous studies, clinical improvement was defined as a decline of two points using a 6-point ordinal scale, similar to this study [2,13]. Wang et al. and Grein et al. demonstrated that the clinical improvement rate on the 28th day after RDV administration was 65.0% and 67.9%, respectively [2,13], which are lower than the rate of this study. This is due to the inclusion of critically severely ill patients (i.e., category 4 or 5) in their studies. In another study, Spinner et al. evaluated the efficacy of RDV assessed by clinical improvement defined as a decline of two points using a 7-point ordinal severity scale, which is the origin of the 6-point scale, in hospitalized patients with mild to moderate severity of disease, and the clinical improvement rate at day 14 was 76.6% (294/384) [14]. It is unclear why the clinical improvement rate was lower compared to the results of our study. One possible explanation is that their study population included more patients with cardiovascular disease or DM as an underlying disease, which are risk factors for disease progression, in comparison to our study. Our results suggest that clinical improvement in RDV treatment may vary depending on the prevalence of aggravation risk factors for COVID-19.

RDV was highly effective in COVID-19 patients requiring oxygen therapy in this study. However, on the 14th day after RDV administration, four patients (11.8%) did not achieve a 2-point clinical improvement (Table 2). For example, patients II and IV had high BMIs (≥25 kg/m^2^), which, in Japan, is defined as obese; they had DM as an underlying disease (risk factor for disease progression [15,16]), and spent more than 20 days in the hospital until they clinically improved by 2 points. This result suggested that COVID-19 patients, who are at high risk of disease progression, may be less effectively treated with RDV. In contrast, patient III showed an early improvement when compared with patients II and IV, despite being obese (BMI = 35.2 kg/m^2^). This may have been due to the fact that the period from symptom onset to RDV initiation was shorter when compared with patients II and IV, consistent with previous reports [17,18,19]. Additionally, Gottlieb et al. showed that the early initiation of RDV in COVID-19 patients with risk factors decreased the composite of COVID-19-related hospitalization or all-cause mortality [20]. Further studies investigating clinical efficacy mechanisms in patients with RDV treatment are warranted.

In terms of RDV safety, 8.5% (4/47) of patients reported CTCAE Grade 3 adverse effects, but no clinically serious adverse events were observed. Tsuzuki et al. reported that 8.4% (69/824) of patients reported mild adverse events, which required no treatment or presented no symptoms [21], consistent with our results. For patient D (Table 3), RDV treatment for 5 days was prioritized, but neutrophil counts increased to 935/µL on the second day after the end of the RDV treatment and returned to baseline (3266/µL) 2 days later. This case was considered as having probable relevance to RDV using the Naranjo scale (score of 6). Neutropenia side effects due to RDV are very rare [21]. As Japanese law requires drug manufacturers and medical personnel to report drug-induced side effect cases, we reported this case (patient D) to the Minister of Health, Labor and Welfare. Since adverse event reports were based on researcher decisions and may therefore be underestimated, further studies are required to explore adverse events during RDV therapy.

Our study had some limitations. Firstly, we used a small sample size in a single institution; therefore, our findings cannot be extrapolated to other hospitals or countries. This study included only COVID-19 patients hospitalized during the fifth infection wave period in Japan (between June and October 2021). Since the homogeneity of the efficacy of RDV on COVID-19 by each virus strain has not been proven in vivo, this study focused on the period when the same strain (i.e., delta variant) was considered to be the cause of infection. Takashita et al. demonstrated that RDV suppresses the growth of not only the delta variant but also the omicron variant in cultured cells, suggesting the efficacy of RDV in patients infected with the omicron variant [22]. Multicenter studies are needed to obtain a larger number of patient samples regardless of SARS-CoV-2 strain type. Secondly, the study was designed as a single arm, similar to previous studies of tocilizumab [23,24,25], and we did not examine clinical factors related to clinical improvements or adverse events in COVID-19 patients receiving RDV treatment. A comparative study using placebo and other antiviral drugs is needed to clarify the factors associated with the efficacy and safety of RDV. Thirdly, the clinical background was not sufficiently investigated in this study. For example, an echocardiographic parameter such as right ventricle function can be useful to characterize myocardial injury and to guide management in patients with COVID-19 [26].

## 5. Conclusions

RDV may be highly effective, with good safety profiles, in mildly to moderately ill patients with COVID-19, especially in patients requiring oxygen therapy. However, further studies are warranted to determine the factors associated with the efficacy and safety of RDV treatment.

## Figures and Tables

**Table 1:** Comparisons of clinical characteristics between non-oxygen therapy group (*n* = 13) and oxygen therapy group (*n* = 34).

Characteristics		Non-Oxygen Therapy (*n* = 13)	Oxygen Therapy (*n* = 34)	*p*-Value
Age (years) ^#^		46 (41–63)	51 (43–57)	0.6856
	≥65 year	3 (23.1)	5 (14.7)	0.6664 ^a^
Gender	Male	6 (46.2)	21 (61.8)	0.3329
	Female	7 (53.9)	13 (38.2)	
BMI (kg/m^2^) ^#^		24.6 (20.6–27.7)	25.2 (22.1–28.0)	0.6428
	<25	8 (61.5)	16 (47.0)	0.8097
	25–30	4 (30.8)	14 (41.2)	
	30<	1 (7.7)	4 (11.8)	
Prior vaccination		1 (7.7)	5 (14.7)	>0.9999 ^a^
Current smoking		3 (23.1)	12 (35.3)	0.5033 ^a^
Comorbidities				
	Hypertension	3 (23.1)	7 (20.6)	>0.9999 ^a^
	DM	0 (0)	4 (11.8)	0.5639 ^a^
	Dyslipidemia	1 (7.7)	1 (2.9)	0.4810 ^a^
	Cerebrovascular diseases	0 (0)	1 (2.9)	>0.9999 ^a^
	Asthma	0 (0)	3 (8.8)	0.5502 ^a^
	Malignant tumor	2 (15.4)	1 (2.9)	0.1812 ^a^
Laboratory findings ^#^				
	WBC (10^3^/µL)	3.9 (2.9–4.8)	5.2 (3.5–5.6)	0.0373
	RBC (10^3^/µL)	444 (415–478)	477 (445–516)	0.0363
	Hb (g/dL)	13.4 (11.3–14.5)	15.0 (13.6–15.7)	0.0109
	Plt (10^4^/µL)	16.9 (13.6–23.7)	17.9 (16.0–22.4)	0.3949
	TP (g/dL)	7.2 (6.9–7.7)	7.4 (7.1–7.7)	0.3162
	ALB (g/dL)	3.6 (3.4–3.8)	3.5 (3.4–3.6)	0.3786
	AST (U/L)	32 (27–41)	41 (31–57)	0.1006
	ALT (U/L)	26 (19–35)	33 (20–57)	0.2952
	LDH (U/L)	258 (197–341)	348 (285–501)	0.0163
	CK (U/L)	90 (48–193)	103 (74–173)	0.5338
	ALP (U/L)	63 (50–76)	69 (58–84)	0.5841
	γ-GT (U/L)	26 (16–54)	65 (25–87)	0.0825
	BUN (mg/dL)	10 (9–12)	12 (10–14)	0.0707
	Cre (mg/dL)	0.63 (0.56–0.90)	0.79 (0.60–0.95)	0.4049
	eGFR (mL/min)	82.3 (67.4–92.5)	76.0 (61.4–91.1)	0.4904
	CRP (mg/dL)	4.27 (0.66–8.54)	5.22 (3.79–7.86)	0.2344
Concomitant drug				
	Dexamethasone	7 (53.9)	34 (100)	0.0002 ^a^
	Baricitinib	0 (0)	7 (20.6)	0.1660 ^a^
	Heparin	0 (0)	7 (20.6)	0.1660 ^a^
	Antibiotics	1 (7.7)	3 (8.8)	>0.9999 ^a^

^#^ Values are expressed as medians and interquartile ranges. ^a^ Fisher’s exact test. ALB, albumin; ALT, alanine aminotransferase; AST, aspartate aminotransferase; ALP, alkaline phosphatase; BMI, body mass index; BUN, blood urea nitrogen; CK, creatine kinase; Cre, creatinine; CRP, C-reactive protein; DM, diabetes mellitus; eGFR, estimated glomerular filtration rate; γ-GT, γ-glutamyl transpeptidase; Hb, hemoglobin; LDH, lactate dehydrogenase; Plt, platelet; RBC, red blood cell; TP, total protein; WBC, white blood cell.

**Table 2:** Clinical characteristics of patients who did not achieve a 2-point clinical improvement on the 14th day of RDV administration in oxygen therapy group.

Patient	Age (Years)	Gender	BMI (kg/m^2^)	Underlying Diseases	Symptom Onset to RDV (Days)	Duration of RDV Treatment (Days)	Concomitant Drug	RDV Initiation to Clinical Improvement by 2 Points (Days)
I	51	Female	18.1	None	9	5	Antibiotics	14
II	56	Male	25.5	DM	8	10	Baricitinib Heparin Antibiotics	24
III	59	Female	35.2	None	4	5	None	15
IV	43	Male	25.7	DM	6	10	Baricitinib Heparin	22

DM, diabetes mellitus; RDV, remdesivir.

**Table 3:** Clinical characteristics and CTCAE Grade 3 adverse effects in COVID-19 patients on RDV treatment.

Patients	Age (Years)	Gender	BMI (kg/m^2^)	Underlying Diseases	RDV Treatment (Days)	Concomitant Drug	Oxygen	Adverse Events	Change in Value ^a^
A	54	Male	26.1	None	5	Dexamethasone	No	ALT (U/L) elevation	Day 1: 32 Day 5: 172
B	45	Male	25.1	None	5	Dexamethasone	Yes	ALT (U/L) elevation	Day 1: 38 Day 8: 200
C	47	Male	21.5	None	5	Dexamethasone	Yes	ALT (U/L) elevation	Day 1: 31 Day 6: 193
D	40	Female	20.7	None	5	None	No	Neutropenia (/µL)	Day 1: 3432 Day 3: 364

^a^ Laboratory test values on indicated days after RDV initiation. ALT, alanine aminotransferase; BMI, body mass index; RDV, remdesivir.

## Data Availability

Not applicable.
